# Severe asthma: prescribing criteria and asthma control test improvement

**DOI:** 10.1186/1939-4551-8-S1-A96

**Published:** 2015-04-08

**Authors:** Sérgio Duarte Dortas, Katya Alves De Sousa, Mauricio Barbosa Fonseca, Annanda Da Silva Aguiar, Monica Flores Rick, Leandro Vianna, Maria Das Graças Basilio Rios, Priscila Sepulveda

**Affiliations:** 1 Hospital Universitário Clementino Fraga Filho Hucff-Ufrj, Brazil; 2 Universidade Iguaçu, Brazil; 3 Centro De Referencia Para o Tratamento Da Asma De Difícil Controle, Brazil

## Background

Severe asthma are linked with high morbidity, significant mortality and high treatment costs. Omalizumab has been shown to decrease the risk of hospitalization or Emergency Department (ED) visits in patients with uncontrolled severe allergic asthma. We aim to describe the conditions under Omalizumab was prescribed in patients followed in a Reference Center for Severe Asthma Treatment in Nova Iguaçu, Rio de Janeiro; and assess the effects of Omalizumab through the Asthma Control Test (ACT) in those patients who had at least a 16 week course.

## Methods

Asthmatic patients treated with omalizumab between February 2013 and June 2014 were evaluated retrospectively. The conditions under Omalizumab was prescribed and ACT improvements were evaluated.

## Results

A total of 19 patients (14 females and 5 males) were prescribed omalizumab. Prescribing criteria were: one or more ED visits in the last year (100%); high dose inhaled corticosteroid and long-acting beta2agonist use (94.7%); systemic corticosteroid use more than 3 times the last year (89.5%); FEV<80% (78.9%); daily short-acting beta2agonist use (68.4%); fast pulmonary function deterioration after systemic corticosteroid withdrawal (52.6%); death threatening asthma exacerbation episode (42%). Seven of these patients had a 16 week course of omalizumab with a significant improvement in ACT total score in six of them (86%).

## Conclusions

In our casuistic, the main criteria omalizumab was prescribed for severe asthma was ED visits. Omalizumab promoted a significant improvement in most patients' ACT total score.

